# Conserved Sequence Processing in Primate Frontal Cortex

**DOI:** 10.1016/j.tins.2016.11.004

**Published:** 2017-02

**Authors:** Benjamin Wilson, William D. Marslen-Wilson, Christopher I. Petkov

**Affiliations:** 1 Institute of Neuroscience, Henry Wellcome Building, Newcastle University, Framlington Place, Newcastle upon Tyne, UK; 2 Centre for Behaviour and Evolution, Henry Wellcome Building, Newcastle University, Framlington Place, Newcastle upon Tyne, UK; 3 Department of Psychology, University of Cambridge, Cambridge, UK

**Keywords:** cognition, language, neurobiology, human, monkey, evolution

## Abstract

An important aspect of animal perception and cognition is learning to recognize relationships between environmental events that predict others in time, a form of relational knowledge that can be assessed using sequence-learning paradigms. Humans are exquisitely sensitive to sequencing relationships, and their combinatorial capacities, most saliently in the domain of language, are unparalleled. Recent comparative research in human and nonhuman primates has obtained behavioral and neuroimaging evidence for evolutionarily conserved substrates involved in sequence processing. The findings carry implications for the origins of domain-general capacities underlying core language functions in humans. Here, we synthesize this research into a ‘ventrodorsal gradient’ model, where frontal cortex engagement along this axis depends on sequencing complexity, mapping onto the sequencing capacities of different species.

## Trends

Cross-species studies of sequence processing are providing insights into combinatorial learning in nonhuman animals that may represent evolutionary precursors to human language-related operations.

Recent comparative neuroimaging studies in humans and monkeys provide neurobiological evidence for evolutionarily conserved sequencing processes, supported by functionally homologous subregions of frontal cortex in humans and monkeys.

These findings point to further development of animal model systems in which the neuronal mechanisms that support these operations can be studied in ways not feasible in humans.

A heuristic ‘ventrodorsal gradient’ model is proposed of primate frontal cortex engagement that depends on sequencing complexity.

## Relationships between Sequence Processing, Primate Cognition, and Human Language

Human language is an unrivalled mode of communication. It reflects our vast combinatorial capacity to generate and recognize an infinite number of novel communicative sequences, combining syntactic knowledge (rules of language) with semantic labels (words and their meanings). Viewed as an evolved neurobiological system, language depends on neurocognitive processes and neural substrates that may be specific to the domain of language (**domain-specific**; see Glossary) or require access to more **domain-general** neurocomputational capacities. An explanatory dissection of these systems will require a multilevel, cross-species approach. Language-specific and domain-general processes can be compared and contrasted in humans 1, 2, while cross-species comparisons can identify which domain-general capabilities are evolutionarily conserved 3, 4, 5, 6, 7, 8 and thereby which functionally conserved neurobiological processes support human behavior 9, 10. Here, we focus on the sequential nature of linguistic communication and the relationship of this to sequence processing as a conserved but variable capacity across the Primate order.

**Figure 1:**
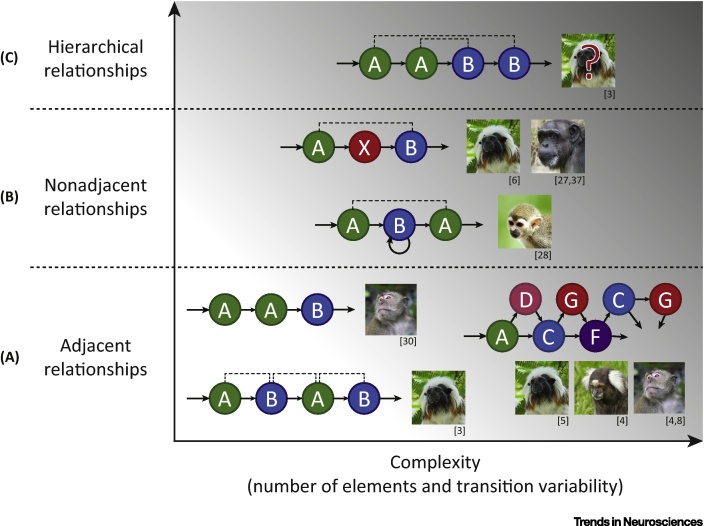
Taxonomy of Sequencing Complexity and Primate Abilities. Relationships between events in a sequence can vary in complexity on multiple dimensions (Box 1, main text). (A) One dimension of complexity (Y-axis) that is relevant for language begins with the capacity to evaluate adjacent relationships (or dependencies) between items in a sequence. This requires an incoming element to be at least temporarily held in memory, compared and associated with the next element in the sequence. (B) Another level of complexity, which human infants learn during the first year of life [6], is processing nonadjacent relationships. In this case, a relationship must be learned between two elements separated in time. For example, nonhuman primates have been shown to learn that the first element in a three element-long sequence predicts the final element, while ignoring an intervening element that is uninformative about the nonadjacent relationship between the first and third element [6]. Moreover, squirrel monkeys were able to learn that the pitch of the first tone in a sequence predicted the final tone, separated by a variable number of tones of a different pitch [28]. This requires holding a stimulus in working memory and comparing it to another element over one or more intervening elements so that the nonadjacent dependency can be established, assessed, or monitored. (C) Hierarchical relationships ​, prominent in language, reflect even higher levels of complexity, such as one phrase (e.g., ‘AB’) being nested inside of another ‘AB’ phrase, requiring multiple ‘A-B’ associations to be simultaneously held in memory. Relative to this taxonomy of sequencing complexity, some currently known sequence processing capabilities of nonhuman primates are illustrated (see the section ‘Sequence Learning: A Candidate Language Precursor’ in the main text). Question marks denote experiments in which species showed no evidence of sensitivity to certain types of sequencing violations (see [3] for details), indicating uncertainty as to whether these species are able to process sequences at the given level of complexity (see Outstanding Questions, main text).

Structured sequence-learning tasks, including statistical learning and **artificial grammar**-learning paradigms, can be used to determine whether an individual is sensitive to different types of ordering relationships between items in a sequence (Figure 1). Performance on these tasks has been shown to correlate with performance on language tasks 11, 12, 13, engages brain areas within the human language network 14, 15, and is impaired in patients with agrammatic aphasia 13, 16, 17. This evidence indicates that sequencing operations share neural mechanisms with language-related processes in the human brain. Shared sequence-processing capabilities have been identified behaviorally in human and nonhuman animals and sequence processing has been proposed as a potential precursor to language syntax (e.g., 18, 19). Moreover, recent **comparative neuroimaging** experiments have provided novel evidence for evolutionarily conserved, functionally homologous neural substrates for sequence processing in humans and monkeys 9, 10, 20.

Here, we review the comparative behavioral and neuroimaging studies of sequence learning in humans and monkeys and propose a heuristic model describing the involvement of different regions of frontal cortex, within a distributed network of regions, in processing increasingly complex sequencing relationships. Testing this model will require further research (see Outstanding Questions). The recent identification of evolutionarily conserved neural substrates provides critical evidence that structured sequence-learning tasks can provide important insights into how language evolved and identify which specific conserved neural processes related to language can be unraveled mechanistically in animal models. More generally, this research also has implications for understanding how the brain supports complex behaviors, such as the ability to perceive the order of temporal relationships in the world [21].

## Sequence Learning: A Candidate Language Precursor

Most nonhuman animals do not organize their vocalizations into complex structured sequences in the same way as humans, songbirds, and a few other species [22]. Some monkeys are capable of combining their vocalizations in functionally meaningful ways [23], but the combinatorial operations involved are minimal. Furthermore, it is clear that the vocal production systems of extant nonhuman primates differ considerably from the complex sensorimotor systems underpinning speech articulation and combinatorial phonology in the modern human (e.g., 24, 25). However, differences in these vocal output capacities do not exclude that nonhuman primates (and other mammals) may have substantial perceptual capacities for assessing the temporal relationships between environmental events [26]. In this regard, investigative paradigms originally developed to study the sequence-learning capabilities of human adults and infants have been successfully adapted to study similar capacities in a range of nonhuman species, including rodents, songbirds, and a variety of primates 3, 4, 5, 6, 7, 8, 27, 28, 29, 30, 31, 32, 33. In these tasks, typically using artificial grammar-learning paradigms, participants are first exposed to exemplary sequences of stimuli that contain sequencing regularities or dependencies between the elements in the sequence (sounds, pictures, etc.). The participants are then tested with novel sequences that either follow or violate these regularities to assess which ordering dependencies they can learn and what strategies they might use to do so. The complexity of such dependencies can be parametrically varied to study more or less language-like ordering relationships 4, 34 (Figure 1).

A relatively simple sequencing operation, for example, might involve recognizing relationships between two adjacent items in a short sequence (e.g., [3] in Figure 1A), which many species are capable of learning 4, 5, 6, 8, 9, 27, 28, 29, 30, 35. However, even with adjacent relationships ​, demands on learning and memory can increase as greater variability is introduced - for example, when the transitional probabilities between items in longer sequences become less predictable (as illustrated along the X-axis in Figure 1 4, 5, 8). Nonadjacent relationships generate a further increase in sequencing complexity (Figure 1B). There is accumulating evidence that some animals are sensitive to temporally separated, nonadjacent sequencing relationships ​^6^, ^28^, ^29^, ^35^, ^36^, ^37^, in which an item must be held in working memory over time for these relationships to be recognized (Figure 1B).

Hierarchically organized sequences, containing nested or recursive relationships between items, have been widely argued to reflect more language-like structures 3, 38, 39 (Figure 1C [3]). However, despite several studies testing whether birds or monkeys can process these more complex sequences 3, 27, 33, 40, it remains unclear whether nonhuman animals can learn such relationships 32, 41. While this leaves unresolved the ultimate limits of nonhuman sequence-processing capabilities and where the cognitive capabilities of humans and other animals may diverge 42, 43 (see Outstanding Questions), the current artificial grammar-learning literature demonstrates that several species have significant capabilities in this domain, involving both adjacent and nonadjacent sequencing operations 4, 5, 6, 8, 9, 27, 28, 29, 30, 35, 36, 37. The neural substrates supporting these capacities can now be studied across species using neurobiological techniques.

## Sequence Processing in the Primate Brain: Testing Neuroevolutionary Hypotheses

In humans, sequence-learning and processing tasks can engage similar regions of frontal cortex to those involved in processing natural language 14, 15. The level of engagement of frontal regions depends critically on the complexity of the sequencing operations 39, 44, 45, with adjacent sequencing operations involving more ventral frontal regions, such as the frontal operculum, while more complex nonadjacent or hierarchically structured relationships also involve **Brodmann Areas** 44/45 (BA 44/45) 14, 45, including Broca's area in the left hemisphere.

Friederici and colleagues hypothesized that the function of the frontal operculum might be evolutionarily conserved, serving a homologous role in processing adjacent sequencing relationships in humans and extant nonhuman primates (**frontal operculum hypothesis** 39, 46). By contrast, BA 44 and BA 45 appear (at least in part) to be functionally specialized for linguistic operations not present in nonhuman primates, even though area 44 in monkeys is known to be cytoarchitectonically comparable to BA 44 in humans [47]. A second, complementary evolutionary hypothesis suggests that human language and communication are supported by two distinct but interacting neurobiological systems [48]. Specializations for core syntactic language functions are thought to depend on a left-lateralized frontotemporal system. This system is functionally integrated with an evolutionarily older, more bilaterally distributed network, which is proposed to have more general language-related communicative functions and is shared with nonhuman primates (**dual neurobiological language systems hypothesis** [49]). Recent comparative neuroimaging studies are beginning to test these hypotheses and to ask whether similar sequence-processing behaviors are supported by the same or different neural substrates in humans and other primates.

We first consider oddball sound detection paradigms, which provide an important point of reference for the sequence-ordering operations that we consider later. The neural processing of oddball sounds has been investigated in depth in both human and nonhuman animals (e.g., 50, 51). Two recent studies used deviance detection paradigms to determine how the brains of rhesus macaque monkeys respond either to unexpected oddball sounds or to the absence of an expected sound in a sequence 10, 20. In this paradigm, a standard sequence of ‘A’ tones is infrequently interrupted by a deviant ‘B’ tone at a different pitch (i.e., ‘AAAB’). In both humans and monkeys, fMRI studies show that oddball sound detection engages bilateral regions around auditory cortex 20, 52. Additionally, humans and monkeys listened to a repeated standard string of the form ‘AAAB’, and infrequently heard the deviant string ‘AAAA’, where the terminal ‘B’ item was substituted with an ‘A’. In macaques, this paradigm engaged a broad set of brain areas, including bilateral anterior insula and ventrolateral prefrontal cortex, including area 6vr (immediately posterior to area 44) 10, 20. In humans, the same sequencing processes also engaged ventral and posterior regions of inferior frontal gyrus (IFG), also extending to BA 44 [10]. This suggests that these types of operation engage comparable processes in certain regions of ventral frontal cortex in both species, but with activations in humans extending into BA 44. BA 44 in humans was activated not only by the ‘AAAB’ versus ‘AAAA’ comparison, but also by further contrasts comparing sensitivity to the number of items in the sequences (i.e., ‘AAAB’ versus ‘AB’ or ‘AAAAAB’). By comparison, the monkey fMRI results showed engagement of a ventral frontal set of regions for the sequencing operations and a functionally separate, more dorsal frontal, set of areas for numerosity processing [10]. The authors argued that regions of human inferior frontal cortex (including BA 44) are specialized to integrate different types of operations (in this case sequencing and numerosity), while aspects of these tasks are subserved by segregated processes in nonhuman primates [10].

**Figure 2:**
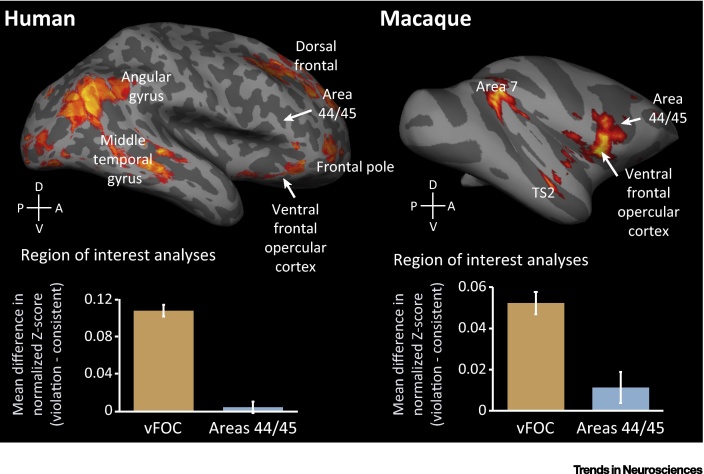
Evolutionarily Conserved Brain Areas for Processing Adjacent Sequence Relationships in Human and Monkey Frontal Cortex. Humans and rhesus macaques were first exposed to exemplary sequences from an artificial grammar that generates variable sequences, based on several adjacent relationships[9] (bottom right of Figure 1, main text). The participants were then presented with ‘consistent’ and ‘violation’ testing sequences during fMRI scanning. Group results from 12 human participants are displayed alongside representative results in an individual macaque, from the three that were studied (for details, see [9]). The results showed voxels in the brain that responded more strongly to violation sequences than to consistent sequences, shown here on rendered lateral surface representations of the human and monkey brain. The effects are illustrated for the right hemisphere, but are not significantly lateralized [9]. A key result was the strong engagement of ventral frontal and opercular cortex (vFOC) in both monkeys and humans, including the frontal operculum and anterior insula. These findings highlight the role of these regions in processing adjacent sequencing relationships. Areas 44 and 45 were not strongly engaged in either species, although the effect was statistically more pronounced in monkeys than in humans. These general impressions are supported by independent neuroimaging evidence in monkeys and humans (see the section Sequence Processing in the Primate Brain: Testing Neuroevolutionary Hypotheses’ in the main text). Parietal activation was observed in both species, including area 39 in humans and area 7 in macaques. These regions form part of the dorsal processing pathway and, in humans, are involved in a range of language tasks, including sentence comprehension 14, 81, 82, 83, but are not thought to form part of the core perisylvian circuit for hierarchical syntax [84]. Involvement of parietal regions is less evident than the involvement of frontal cortex during sequence processing in humans (e.g., 39, 61) and is not consistently observed in monkeys 9, 10.

While oddball tasks typically depend on the detection of an unexpected element in a stream of repeated stimuli, artificial grammar-learning paradigms (as discussed in the previous section) require learning specific rule-based ordering relationships ​, designed to emulate the types of sequential dependencies found in syntactically structured linguistic sequences. The neural substrates underpinning the processing of variable adjacent sequencing dependencies were assessed in a recent comparative human and macaque monkey fMRI study [9]. An artificial grammar [5] was used to generate sequences of nonsense words, containing a range of pairwise transitions between the elements 4, 8, 9. Following exposure to sequences consistent with the artificial grammar, macaque monkeys and human participants showed similar behavioral sensitivity to the adjacent dependencies in the sequences 4, 8. Both species were then scanned while listening to consistent sequences and to sequences that violated the learned ordering relationships ​[9]. In both humans and monkeys, the sequence violations most consistently engaged ventral regions of frontal cortex, including the frontal operculum and anterior insula (Figure 2). While other perisylvian regions were also involved, such as parietal area 7 in monkeys, these were not as reliably activated in the macaques as the ventral frontal and opercular regions. This suggests that these frontal regions are functionally conserved in processing local (adjacent) sequencing dependencies, providing initial support for the frontal operculum hypothesis [39]. However, the effects were not strongly lateralized, which is consistent with the dual neurobiological systems hypothesis, which proposes that the human left-lateralized language system differentiated from a conserved bilateral system that is shared with our primate relatives [49].

Despite the number and variety of transitions that must be processed to detect the ordering violations (arguably an increase in cognitive demands over more predictable adjacent sequencing regularities; Figure 1), this experiment did not strongly engage BA 44/45 in the majority of the human participants (Figure 2). By comparison, the activation in the corresponding anatomical areas 44/45 in macaques, although weaker and more variable than the key observations involving the frontal operculum, was stronger than in humans (Figure 2). The central result of these comparative neuroimaging studies is that key regions of frontal cortex (frontal operculum, insula, and adjacent areas in the ventrolateral prefrontal cortex, VLPFC) may share evolutionarily conserved functions for processing adjacent sequencing dependencies. The role of areas 44 and 45 in such operations in humans and monkeys remains less clear.

## Ventrodorsal Gradient Model of Frontal Cortex Function for Sequencing Operations

Evidence for an evolutionarily conserved, domain-general system is beginning to emerge from the comparative neuroimaging studies of sequence processing in humans and monkeys. To date, the comparative fMRI studies point to a conserved, bilateral, ventral frontal and opercular subsystem within frontal cortex that supports the evaluation of adjacent sequencing dependencies. In both humans and monkeys, ventral regions of frontal cortex, including the frontal operculum and insula, appear to conduct ‘online’ processing of adjacent sequencing dependencies 9, 10, 39 (Figure 2). These areas of VLPFC are also involved in allocating attention to processes of immediate interest 53, 54, 55, 56, and may be particularly involved in processing salient aspects of sensory sequences, such as the first and last items in a sequence [57] or adjacent ordering relationships [58]. By contrast, processing the relationships between more distant or hierarchically structured sequence elements requires predictions to be tracked over time, bringing additional cognitive demands. These types of sequencing operation engage more dorsal areas, such as BA 44 and 45 in humans 15, 39, as well as dorsolateral prefrontal cortex (DLPFC; e.g., BA 46 or 9) when working memory demands increase 35, 57, 59, 60, 61. We suggest that these functions of VLPFC and DLPFC are evolutionarily conserved in human and nonhuman primates for specific sequence-processing operations, driven by the complexity of the ordering relationships ​ involved (e.g., Figure 1 4, 62) and the cognitive operations that these require. Human specialization in these regions for language-related functions is likely to be reflected in more left-lateralized processing for core syntactic analyses [49], differential engagement of areas such as left BA 44/45 for complex sequencing operations 9, 62, 63, and the emergence of a network of regions with greater levels of interconnectivity with other brain areas [64].

**Figure 3:**
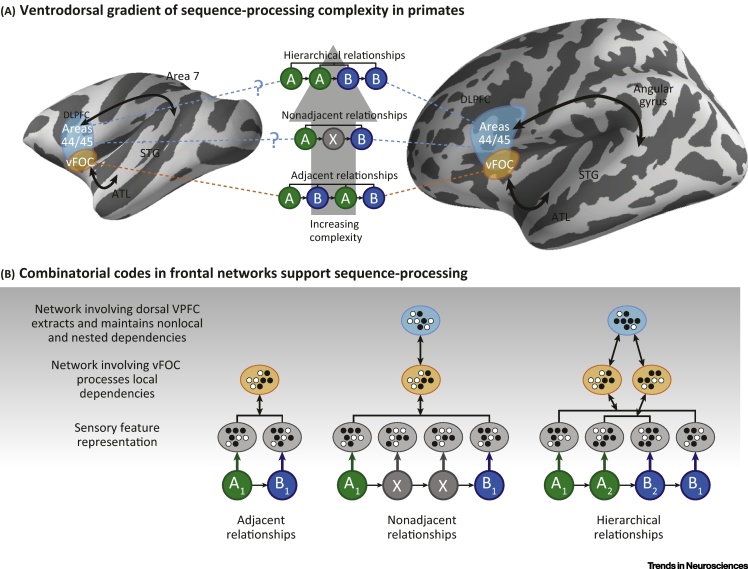
Key Figure: A Gradient of Frontal Network Engagement as a Function of Sequencing Complexity

We propose a ‘ventrodorsal gradient’ model (Figure 3, Key Figure) whereby posterior PFC along this axis supports sequencing operations of increasing complexity in human and nonhuman primates. In monkeys, as in humans, we predict that, within a broad spatially distributed brain network, parts of which we consider below, more extensive involvement of dorsal aspects of VLPFC, including areas 44 and 45, will depend on the complexity of sequencing operations. In relation to the taxonomy of sequencing complexity (Figure 1), we illustrate the sequencing relationships that require processing and where associations need to be established, proposing as we do so how this might affect neural representations (Figure 3): (i) sensory cortex extracts the features of the items that are being processed, and interacts with regions such as the hippocampus 65, 66, 67, cerebellum 68, 69, and frontal cortex to encode and establish sequence-ordering associations. Interactions between neurons in different regions are facilitated by phase-locked neural activity 70, 71; (ii) ventral frontal and opercular cortex, within a broader network that is interconnected with other temporal lobe regions via ventral processing pathways (relying on the uncinate fasciculus and the extreme capsule fibre system) 72, 73, has an evolutionarily conserved function in primates, supporting the analysis of adjacent dependencies. In processing such relationships, this network will generate sequence-order **prediction errors** when incoming input does not match previously learned regularities [9] (Figure 3 [70]); (iii) when sequencing demands require establishing and evaluating more complex relationships ​(e.g., nonadjacent relationships ​ or more complex dependencies, should nonhuman primates be shown to learn these), ventral frontal and opercular regions interact with dorsal and/or anterior aspects of VLPFC (e.g., BA44/45) to extract and monitor for nonadjacent and multiple or hierarchical dependencies. The dorsal processing pathways (arcuate fasciculus in humans and different aspects of the superior longitudinal fasciculus in human and nonhuman primates), that connect inferior frontal and prefrontal regions with temporoparietal cortex 72, 74, 75 allow information about sequencing relationships ​of greater complexity to interact with processing required under different types of cognitive demands or tasks (Figure 3).

Importantly, frontal cortex is not necessarily a storage site for more refined representations, but is conceived here as holding combinatorial codes [76] (Figure 3) that can be used to mediate and integrate different types of sequencing association by recruiting broader aspects of the distributed network. The frontal sites, by feedback to multiple cortical regions, synchronize neural activity patterns corresponding to the feature representations of incoming stimuli and expected stimuli or the associations between stimuli.

Cognitive demands increase as sequences become longer and more complex, and more sequencing relationships ​must be processed simultaneously. It is possible that greater demands on cognitive operations within sequences at a given level of complexity engage more anterior aspects of PFC and/or parts of DLPFC 14, 35, 57, 77, 78. A prominent model of human frontal cortex function defines a posterior-to-anterior axis of frontal involvement for cognitive control. Here, relatively simple tasks engage posterior areas of frontal cortex (including BA 44), while more abstract, hierarchically organized or cognitively more demanding tasks engage anterior frontal regions [78]. For example, stroke patients with lesions in posterior frontal cortex are impaired in processing simpler artificial grammar relationships ​, but not more complex, long-distance relationships ​[79]. More anterior frontal engagement is also observed as sentence complexity increases during second language processing [80]. However, Jeon and Friederici noted that natural language processing, possibly because it is highly automatic in nature, does not necessarily follow this posterior-anterior gradient, and instead engages more posterior regions, including BA 44 and 45 [80].

Comparative work on domain-general sequencing operations and work in humans comparing domain-general and language-specific operations will be required to test the predictions from our ventrodorsal gradient model. Regardless of the outcome, the empirical evidence should shed new light on how frontal cortex has mechanistically differentiated to support language, and which functions are likely to have been evolutionarily conserved.

## Concluding Remarks and Future Perspectives

Unraveling the evolutionary origins of language has challenged scientists and philosophers for centuries. Recent comparative research has made considerable progress, providing new insights and evidence for evolutionarily conserved behaviors and neural substrates. Building on this, our synthesis of recent behavioral and neuroimaging findings in humans and monkeys allows us to propose a model of primate frontal cortex organization that depends on sequencing complexity. The model specifies evolutionarily conserved functions related to sequencing operations in humans and other primates, and it predicts that human frontal cortical regions, such as areas 44 and 45, may have differentiated to support higher-order combinatorial operations that go beyond the sequence-processing capacities exhibited by nonhuman animals. Understanding the extent to which language-related cognitive abilities are conserved will provide a more complete and satisfying account of the evolution of the human language network. Moreover, this approach can assist in the development of better neurobiological models for understanding the neuronal mechanisms of certain aspects of human communication.

Outstanding Questions What are the limits of the sequence-processing capabilities of different animal species? We propose using computational strategies to quantitatively increase sequencing complexity and to identify which sequencing operations can be processed by different species (Box 1, main text). As sequence complexity increases, we expect further behavioral differences across the species to be revealed 3, 4, 7, 76. Different approaches (e.g., using operant training tasks) might identify further ‘hidden’ sequence-processing abilities or allow the assessment of learning strategies.

How is the network of brain areas and neurons that are involved in sequence processing modulated by sequencing operations with higher orders of complexity? In humans, increasingly complex sequencing tasks appear to engage more dorsal regions of the VLPFC (including BA 44 and 45) and brain pathways (arcuate fasciculus) [14]. Ventral regions and pathways may well be largely conserved in nonhuman primates, but which pathways and regions support sequencing operations of greater complexity in nonhuman primates, and how might these relate to those observed in humans?

Are input sequences from different sensory modalities (e.g., auditory and visual) processed by domain-general neural substrates or by modular processes? Although here we have focused on sequences generated by stimuli from one sensory modality, assessing how sequencing operations might hold across sensory modalities can help to further support or clarify notions on domain-general processing.

What are the neuronal mechanisms that underpin sequence-processing capabilities? In animal models, brain areas identified by fMRI, for example, can be probed (recording single-unit activity and neuronal oscillations) and perturbed (e.g., reversible inactivation, microstimulation, or optogenetics) to establish causal relationships ​and provide insights into neural systems and circuit mechanisms.

## Box 1

Quantifying Sequence Complexity

To fully understand the behavioral sensitivities and neurobiological processes that underpin sequence processing, it is necessary to quantify the complexity of different sequence-processing tasks. Computational approaches can facilitate this process 86, 87. Here, we summarize some basic approaches for quantifying sequencing complexity for finite-state systems 4, 19, 88, where nonhuman animal abilities are thought to largely reside.

The statistical dependence between adjacent elements in a sequence can be calculated using transitional probabilities (TP) or mutual information [8]. For example, an ‘A’ item may be followed by a ‘B’ item with known probability, calculated from the transitions in the learning or exposure set of sequences*.* The statistical predictability of pairwise transitions can then be assessed in the testing sequences. Illegal transitions are those that occur with 0 probability and affect the sum or average pairwise TPs of a sequence relative to those that have only legal pairwise transitions. The statistical predictability of a sequence can be used to model and design testing sequences for assessing behavioral data or regressing with neurobiological data.

It may also be important to directly compare the unpredictability or entropy of different artificial grammar structures 4, 88. A simple approach, borrowed from analyses of birdsong complexity [89], is to calculate the nonlinearity of the available transitions. Sequences that are less predictable are cognitively more demanding, regardless of whether the sequences contain adjacent or non-adjacent dependencies.

Nonadjacent relationships ​can, for example, be modeled by higher-order Markov state chains [19] or hidden Markov models [88]. These approaches are sensitive to temporal dependencies over a series of elements. For hierarchically nested relationships, a computational push-down stack can be used to build nested structures, as well as providing a model for the computational and cognitive (memory) operations involved [18]. It remains an open question which computations are most predictive of nonhuman animal behavior under various conditions and how they might be implemented in the brain.

## Glossary

Artificial grammar: rule-based system that is used to generate legal strings that follow specific rule(s). Typically, there is a learning phase, during which the participant is exposed to or trained with legal sequences, followed by a testing phase, where novel legal sequences are presented along with sequences that violate certain rules. Probe ‘violation’ sequences can be designed to provide information about which rules the participants can learn.
Brodmann Area (BA): anatomically defined regions of cortex, originally identified by German anatomist Korbinian Brodmann during the early 1900s, modified and expanded upon by modern neuroanatomists.
Comparative neuroimaging: using the same neuroimaging approach (e.g., fMRI or EEG) in two or more species, to allow direct comparison of functional activations elicited by specific tasks.
Domain general: operations whose function can be applied across multiple cognitive domains. For example, allocating attention to or remembering an item is thought to engage domain-general processes where the same operation is applied to different forms of input. In humans, for example, these can be linguistic or nonlinguistic materials.
Domain-specific: it is thought that certain language operations evolved specifically for the use of language, so that ‘domain-specific’ in this context refers to language domain-specific operations. For example, applying syntactic knowledge to a category of words (nouns, verbs, and adjectives) depends on the grammar of a particular language. Such operations may serve language-specific functions and depend on specialized processes within and adjacent to regions involved in domain-general operations.
Dual neurobiological language systems hypothesis: the left hemisphere-lateralized language system is integrated within an older more bilaterally distributed system, which has more general language-related communicative functions and is proposed to be held largely in common with nonhuman primates [49].
Frontal operculum hypothesis: the frontal operculum is a region of frontal cortex ventral to BA 44 and 45. It includes the opercular dysgranular insula and adjacent regions. The hypothesis originally articulated by Friederici [46] proposes that adjacent sequencing operations engage the frontal operculum in humans and monkeys.
Prediction error: predictive coding models of perception hypothesize that top-down prediction signals from hierarchically higher areas are compared with incoming sensory information from hierarchically lower areas, generating error signals that are fed forward. In terms of sequence processing, perceiving any sequence element should elicit a prediction about the next expected element in the sequence. If an unexpected (violation) element follows, a prediction error will be elicited in sites that process that particular level of sequencing complexity (Figure 3, main text).
