# Supplementary material for: Conserved Sequence Processing in Primate Frontal Cortex
Source: Trends Neurosci. 2017 Feb;40(2):72–82. doi: 10.1016/j.tins.2016.11.004 (PMC5359391; doi:10.1016/j.tins.2016.11.004)
Supplement: Supplementary file 1 [file mmc1.pdf]

**Question 1:**

How might sequence processing abilities be useful for nonhuman animals, who do not have human language as we know it, such as nonhuman primates? Pick the one INCORRECT answer.

❍ Recognizing the relationship between environmental events (relational knowledge), some of which might predict the occurrence of others in time

❍ Combining calls to extend an animal’s behavioural communicative range using a finite set of vocalizations

● To map a sensory stimulus with a motor response (sensory-motor mapping)

*Explanation:*

All the other answers are correct, but sequence learning or processing by definition requires a sequence of events, which goes beyond the mapping of a particular single sensory feature to a motor response.

❍ To recognize whether a vocal exchange between other animals in the wild is consistent with the animal’s prior social knowledge

❍ To plan a pattern of motor movements

**Question 2:**

What aspects of language do artificial grammar learning experiments aim to emulate?

❍ Communicating meaning (semantics)

● Word order relationships

*Explanation:*

Generally artificial grammars emulate pure ordering relationships between sounds or

pictures in a sequence. The grammar (rules) dictates which sequence elements are related to others in the sequence, some of which may be separated in time and thus have non-adjacent relationships. This can be related to ‘phonotactics’ (the organization of phonemes within a word) but is not aiming to emulate the acoustics of phonemes (answer 4, below).

❍ Pragmatic aspects of language

❍ Phoneme acoustics

❍ Compositional syntax (how syntactic structure is used to relay meaning)

**Question 3:**

Name the neuroevolutionary hypothesis that explains the left hemispheric lateralisation effects seen in humans, and the ancestral system that they might stem from

❍ Frontal operculum hypothesis (Friederici and colleagues)

❍ Universal grammar hypothesis (Chomsky and colleagues)

❍ Multimodal nature of language precursors hypothesis (Fitch and colleagues; others)

❍ Asymmetrical temporal processing hypothesis (Poeppel and colleagues)

● Dual neurobiological systems hypothesis (Marslen-Wilson and colleagues)

*Explanation:*

The dual neurobiological systems hypothesis stipulates that syntactic language operations on lexical items involves a left lateralized frontal system. This system works alongside a bi-hemispheric system that appears to be conserved in nonhuman primates, based on the recent comparative studies that the review paper considers. Note that this hypothesis may not be mutually exclusive from the others listed here, but the asymmetrical temporal processing hypothesis focuses on temporal integration windows in the brain and is not specific to syntax and the frontal systems.

**Image 1**

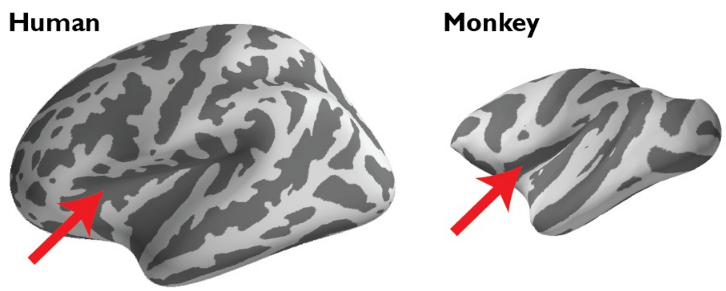


Which brain region is identified by the arrow in these human and monkey brain images from the paper?

**Question 4:**

In which of these ways is comparative neuroimaging most useful?

❍ It helps us to understand language-specific and domain-general operations

❍ It helps us to understand the neurobiology of syntax

● It helps us to understand evolutionarily conserved domain-general operations

*Explanation:*

Only this answer is correct. The other answers point to things that can only be conducted in humans because they rely on human language or in one species (monkeys). Both comparative studies and those in humans are useful because the former helps us to understand which evolutionarily conserved processes can be modelled with animals using approaches not possible for study in humans, and the latter helps us to understand with the tools available in humans which aspects of our neurobiology have specialized specifically for language.

❍ It helps us to understand the semantic representations

❍ It helps us to understand the neurobiology of the monkey brain
